# Information Needs and Counseling Preferences among Potential Users of the Future Teratology Information Service in Belgium: A Cross-Sectional Study Involving the Public and Healthcare Professionals

**DOI:** 10.3390/ijerph19148605

**Published:** 2022-07-14

**Authors:** Michael Ceulemans, Kristel Van Calsteren, Karel Allegaert, Veerle Foulon

**Affiliations:** 1 Clinical Pharmacology and Pharmacotherapy, Department of Pharmaceutical and Pharmacological Sciences, KU Leuven, 3000 Leuven, Belgium; karel.allegaert@kuleuven.be (K.A.); veerle.foulon@kuleuven.be (V.F.); 2 Teratology Information Service, Netherlands Pharmacovigilance Centre Lareb, 5237 MH Hertogenbosch, The Netherlands; 3 L-C&Y, Child and Youth Institute, KU Leuven, 3000 Leuven, Belgium; 4 Department of Obstetrics & Gynecology, University Hospitals Leuven Gasthuisberg, 3000 Leuven, Belgium; kristel.vancalsteren@uzleuven.be; 5 Woman and Child, Department of Development and Regeneration, KU Leuven, 3000 Leuven, Belgium; 6 Department of Clinical Pharmacy, Erasmus MC, 3000 CA Rotterdam, The Netherlands

**Keywords:** pregnancy, breastfeeding, lactation, obstetrics, medication, community health services, public health, drug safety, drug information services, information seeking behavior

## Abstract

A Teratology Information Service (TIS) does not exist in Belgium yet but will hopefully be established soon. To prepare for this, we aimed to provide insight into the information needs and counseling preferences of the Belgian public and healthcare professionals (HCPs) regarding medication use in pregnancy and breastfeeding. A cross-sectional study using two anonymous, online surveys disseminated via social media, websites, and newsletters addressing Dutch and French-speaking individuals (≥18 years) and licensed HCPs was performed between June and September 2020. Ethics approval and informed consent were obtained. In total, 1508 public survey respondents (98% women) and 702 HCPs participated. Information needs on perinatal medication use were ubiquitous among both groups, and for which they often relied on patient information leaflets or the product information and online fora. Conflicting information on this topic regularly occurs and complicates HCPs’ duties. Women and HCPs assigned an important role to a TIS, both in terms of providing evidence-based information (via a website or app) and being accessible to be contacted in case of questions (by phone or via e-mail or chat). In conclusion, a TIS would be warmly welcomed by women and HCPs in Belgium and should ideally be established soon to address current information needs regarding perinatal medication use and to contribute to research in this field.

## 1. Introduction

Pregnancy and breastfeeding are unique situations where the benefits of pharmacotherapy are weighed against potential risks for the woman and her fetus or nursing infant. To make informed decisions, women and health care professionals (HCPs) need reliable information on the safety of medication use during pregnancy and breastfeeding. Besides the overall lack of safety data for most substances [1], inaccurate information and inconsistencies across (online) sources and the product information/patient information leaflet (PIL) are common practice regarding perinatal medication use [2,3,4,5,6]. This may potentially lead to anxiety, medication avoidance, poor medication adherence and/or early breastfeeding cessation [5,7]. Nevertheless, safe use of medication and other products during pregnancy and breastfeeding is critical to safeguard mother–infant outcomes. The potential risk of adverse fetal and neonatal effects associated with some medicines further point to the complexity and challenges associated with medication use in this population [8].

Similar to other countries [9,10], studies in Belgium have shown that the public and HCPs may regularly have questions on medication safety during pregnancy and breastfeeding. In response, they search for answers via multiple and sometimes less expected ways, such as contacting the Poison Center to ask questions without yet being exposed [11,12]. Although a national Teratology Information Service (TIS) is considered appropriate following a statement by the European Board & College of Obstetrics & Gynecology [13], Belgium still has no TIS yet. However, from a public health perspective, and to meet the information needs on perinatal medication use, the establishment of a Belgian TIS was advised to policy makers in 2016 and 2019. A TIS can be considered as an expertise center on the exposure to medicines, health products, illicit drugs, radiopharmaceuticals and infections during preconception, pregnancy, and breastfeeding [14]. TISs’ activities are centralized around three pillars: (1) counseling of HCPs and (possibly) the public by providing evidence-based information on this topic; (2) collecting medication exposure and mother–infant outcome data for research purposes on perinatal medication safety; and (3) contributing to the (continuous) education of licensed/future HCPs. From a research perspective, a TIS entails the opportunity to collect data on medication exposure and outcomes, contributing to the generation of new evidence including signal detection of potential teratogens [15]. A review on the effectiveness of TISs has shown that these services improve mother–infant outcomes and have the potential to lead to healthcare and personal cost savings [16]. TISs have been in place in many countries in Europe and beyond for many years or decades [14,17,18,19,20,21].

While a Belgian TIS may be established in the (near) future, its ‘operational’ requirements remain largely unknown. In other words, there is still a lack of evidence on the content and type of information the public and HCPs in Belgium need, or which role they assign to a TIS in terms of conveying evidence-based information on this topic. Likewise, evidence is missing on the expectations of the public and HCPs towards the accessibility of the TIS, and whether users are willing to contact the TIS for personal counseling purposes in case of specific questions (and if so, in which way).

Therefore, in view of the expected establishment of a Belgian TIS that meets potential users’ needs and expectations, this study aimed to provide insight into the information needs and counseling preferences of the public and HCPs in Belgium regarding medication use during pregnancy and breastfeeding. Such insight is pivotal to set up a service that is adapted as much as possible to and optimally embedded in the Belgian setting.

## 2. Materials and Methods

### 2.1. Study Design and Sample

A cross-sectional study using two online, anonymous (Qualtrics) surveys, addressing both the public and HCPs, was performed in Belgium between mid-June and the end of September 2020. Dutch (NL)- and French (FR)-speaking female and male individuals (≥18 years) and licensed HCPs were eligible to participate. More specifically, pregnant and breastfeeding women (now and/or in the past) and their partners, as well as women and their partners willing to conceive in the (near) future could enroll. Further, HCPs who were involved in some way in the counseling of pregnant and breastfeeding women and couples planning to conceive could participate, such as gynecologists, midwives, medical specialists, pediatricians, neonatologists, general practitioners (GPs), hospital and community pharmacists, nurses, and lactation consultants.

The public survey was promoted via social media accounts and popular websites often visited by women willing to conceive, pregnant women and/or breastfeeding mothers, as well as via perinatal organizations, expertise centers on maternity care, and patient groups. The survey for HCPs was distributed via newsletters and websites of professional organizations of different types of HCPs involved in perinatal care, and through the Belgian Center for Pharmacotherapeutic Information.

Survey participants provided online informed consent prior to survey initiation. Ethical approval for study execution was obtained from the Ethics Committee EC Research UZ/KU Leuven (S64158; 8 June 2020). Study reporting was performed according to the STROBE guidelines [22].

### 2.2. Survey

The surveys for the public and for HCPs were quite similar and consisted of multiple-choice questions exploring (1) information needs on medication use in general (public survey) and during pregnancy and breastfeeding; (2) information preferences regarding the content and format of the information to be provided by a TIS; (3) counseling preferences including accessibility of a TIS, timing of contact, and acceptable waiting time; and (4) characteristics of respondents. Next, the level of confidence among the public towards information on medication use during pregnancy/breastfeeding that would be provided by a TIS was rated on a 5-point Likert scale ranging from full confidence, confidence, neutral, little confidence to no confidence. Finally, HCPs were also asked to indicate their level of agreement with four statements on the availability of information on (perinatal) medication use and the effects of contacting a TIS, rated on a 4-point Likert scale ranging from (totally) agree to (totally) disagree.

The Dutch surveys were pilot-tested by 5 individuals and 5 HCPs (i.e., 1 pharmacist, 1 GP, 1 gynecologist, 1 midwife, 1 nurse/lactation consultant) and were modified accordingly to avoid errors and technical inconsistencies and to improve the clarity of some questions. The final version of the Dutch survey was translated into French by a professional translator.

### 2.3. Data Analysis

Absolute counts (n) and percentages were used to descriptively analyze the survey responses from the public and HCPs. All collected data were retained for the analyses.

Any degree obtained after secondary school was considered ‘higher education’. The frequency of HCPs’ contact with the target population was dichotomized into ‘never/ yearly’ and ‘at least monthly’ (for the latter, the response options monthly, weekly, and daily were taken together). The results of the question on the level of confidence of the public towards information on medication use during pregnancy/breastfeeding provided by the TIS were categorized and presented as the percentage of individuals reporting (full) confidence. The results of the four statements completed by HCPs were dichotomized and presented as the percentage of HCPs (totally) agreeing with each of the statements.

Chi-square tests were performed to determine characteristics associated with willingness to contact the TIS in cases of questions on perinatal medication use. For public survey respondents, the following characteristics were assessed: age (≤30 y or >30 y), relationship status (partner or no partner), professional status (employed or not), education level (higher or lower education), pregnancy experience (yes or no), breastfeeding experience (yes or no), history of assisted reproductive technology (ART) (yes or no), medication use during pregnancy (yes or no), and medication use during breastfeeding (yes or no). For HCPs, the characteristics of gender (female or male), age (<35 y or ≥35 y), and frequency of contact with women/couples willing to get pregnant, pregnant women, breastfeeding women, and (nursing) infants (never/yearly or at least monthly) were assessed. Results were considered significant if *p* ≤ 0.05. The results for the variable profession were only descriptively shown; no chi-square tests were performed due to the very limited sample size for some HCP disciplines. The data were analyzed using SPSS Statistics version 28 (IBM Corp, Armonk, NY, USA).

## 3. Results

### 3.1. Characteristics of Participants

In total, 1508 public survey respondents (NL: n = 1181; FR: n = 327) and 702 HCPs (NL: n = 457; FR: n = 245) completed the survey. Of both groups, 72% and 76%, respectively, answered all questions. The median age of public survey participants and HCPs was 31 years (IQR: 5) and 33 years (IQR: 16), respectively. For both surveys, at least 80% were women, with only a few men participating in the public survey (<2%) (see Table 1 and Table 2).

Most public survey respondents were highly educated (87%) and professionally active (92%), with one-third working in healthcare. While >90% of the female respondents had been pregnant before or were currently pregnant, 75% already had breastfeeding experience (see Table 1). Compared with population data, respondents were higher educated, more often professionally active, and more often engaged in a relationship [23,24,25].

With regard to participating HCPs, most of them were community pharmacists (36%), GPs (21%), hospital pharmacists (14%) or midwives (13%) (see Table 2). Overall, >80% reported professional contact with pregnant and breastfeeding women and (nursing) infants at least each month (see Supplementary Material Table S1). While 48% answered receiving questions on medication use in pregnancy and breastfeeding at least weekly, this happened every month for 87% of the HCPs.

**Table 1:** Characteristics of the public survey participants according to the language of survey completion (N = 1508).

	Total (N = 1508)	Dutch-Speaking Respondents (N = 1181)	French-Speaking Respondents (N = 327)
	% (n)	% (n)	% (n)
**Gender**			
Female	98.1 (1065)	97.7 (850)	99.5 (215)
Male	1.9 (21)	2.3 (20)	0.5 (1)
**Relationship status**			
Partner	93.6 (1017)	94.9 (826)	88.4 (191)
No partner	6.4 (69)	5.1 (44)	11.6 (25)
**Professional status**			
Professionally active	91.7 (989)	94.7 (823)	79.0 (166)
Not professionally active	8.3 (90)	5.3 (46)	21.0 (44)
**Highest education**			
Higher education	87.3 (942)	87.7 (763)	85.6 (179)
No higher education	12.7 (137)	12.3 (107)	14.4 (30)
**Status of female respondents ^1^**			
Currently pregnant	30.5 (324)	33.0 (280)	20.5 (44)
Currently breastfeeding	45.4 (483)	40.0 (340)	66.5 (143)
Trying to get pregnant	8.6 (92)	9.3 (79)	6.0 (13)
None of the above	19.5 (208)	21.4 (182)	12.1 (26)
**Gestational trimester**			
First trimester (0–12 weeks)	24.0 (75)	24.8 (67)	19.0 (8)
Second trimester (13–27 weeks)	30.4 (95)	31.5 (85)	23.8 (10)
Third trimester (28–40 weeks)	45.5 (142)	43.7 (118)	57.1 (24)
**Current breastfeeding duration**			
≤6 weeks	11.8 (57)	13.2 (45)	8.5 (12)
Between 6 weeks–6 months	39.6 (191)	41.8 (142)	34.5 (49)
> 6 months	48.5 (234)	45.0 (153)	57.0 (81)
**Pregnancy history of female respondents**			
Currently pregnant or have been pregnant before	94.6 (1007)	93.5 (794)	99.1 (213)
Have not been pregnant yet	5.4 (57)	6.5 (55)	0.9 (2)
**Breastfeeding history of female respondents**			
Currently breastfeeding or breastfeeding before	74.6 (794)	70.7 (600)	90.2 (194)
No breastfeeding experience	25.4 (270)	29.3 (249)	9.8 (21)
**History of ART among female respondents** **^2^**			
Yes	15.7 (167)	16.0 (136)	14.4 (31)
No	84.3 (897)	84.0 (713)	85.6 (184)
**Medication use among female respondents ^3^**			
At some time during pregnancy	77.6 (781)	77.3 (614)	78.4 (167)
At some time during breastfeeding	77.3 (614)	75.7 (454)	82.5 (160)
In the last 7 days	51.4 (546)	51.5 (437)	50.7 (109)
Current medication use due to a chronic illness	19.2 (204)	18.9 (160)	20.5 (44)

Results are shown as % (n). Numbers may not add up due to missing values. ^1^ the numbers exceed 100% as women could indicate multiple answers. ^2^ ART = assisted reproductive technology. ^3^ Medication use did not include folic acid or multivitamins.

**Table 2:** Characteristics of the participating HCPs according to language of survey completion (N = 702).

	Total (N = 702)	Dutch HCPs (N = 457)	French HCPs (N = 245)
	% (n)	% (n)	% (n)
**Gender**			
Female	80.7 (430)	82.6 (294)	76.8 (136)
Male	19.3 (103)	17.4 (62)	23.2 (41)
**Having children**			
Yes	56.5 (301)	55.3 (197)	58.8 (104)
No	43.5 (232)	44.7 (159)	41.2 (73)
**Currently willing to have children**			
Yes	32.1 (171)	36.5 (130)	23.2 (41)
No	67.9 (362)	63.5 (226)	76.8 (136)
**Profession**			
Community pharmacist	35.8 (191)	35.1 (125)	37.3 (66)
General practitioner	20.6 (110)	19.9 (71)	22.0 (39)
Hospital pharmacist	13.9 (74)	11.5 (41)	18.6 (33)
Midwife	13.3 (71)	17.7 (63)	4.5 (8)
Pediatrician/neonatologist	4.5 (24)	3.1 (11)	7.3 (13)
Lactation consultant	3.8 (20)	4.2 (15)	2.8 (5)
Gynecologist	3.4 (18)	4.8 (17)	0.6 (1)
Medical specialist	3.0 (16)	3.4 (12)	2.3 (4)

Results are shown as % (n). Numbers may not add up due to missing values.

### 3.2. Medication Information Needs and Seeking Behavior of the Public

Overall, 94% of the public respondents answered having already searched for general information on medication use. The main sources of information were the patient information leaflet (PIL) (95%), Google or other search engines (83%), websites/social media of (online) pharmacies (24%), scientific resources (23%) and online fora (19%) (see Supplementary Material Table S2). The main reasons for searching were to obtain more information on side effects (63%), to check received information (58%) and to facilitate making decisions about personal health (47%). Importantly, having received insufficient (34%) or conflicting information from HCPs (25%) were also regularly cited by respondents (see Supplementary Material Table S3).

Moreover, 92% and 68% of the public respondents reported having already searched for information on medication use during pregnancy or breastfeeding, respectively. A large difference was observed between women with or without previous pregnancy experience (95% vs. 54%) and women with or without previous breastfeeding experience (86% vs. 15%). Main sources of information on medication use in pregnancy/breastfeeding were the PIL (90%; 86%), Google or other search engines (77%; 70%), online fora (25%; 23%), scientific resources (22%; 23%), and websites/social media of perinatal organizations (21%; 21%) (see Supplementary Material Table S2). The official government-funded website containing evidence-based information on healthy pregnancies (www.gezondzwangerworden.be (accessed on 19 June 2022)), was known by 16% and had been visited by 15% of the public respondents. The main reasons for searching were wanting to have more information on the safety of a medicine for the fetus/nursing infant (67%) or for herself as pregnant/breastfeeding woman (54%), to check received information (50%), to have more information on side effects (38%), and to facilitate making decisions about personal health (34%). Having received conflicting (27%) or insufficient information from HCPs (25%) were also regularly mentioned (see Supplementary Material Table S3).

### 3.3. Medication Information Needs and Seeking Behavior of HCPs

Overall, 85% of HCPs indicated searching for information on medication use during pregnancy/breastfeeding at least monthly. To find information, HCPs often check the product information, followed by discussions with colleagues from the same profession, checking guidelines on the treatment of conditions during pregnancy/breastfeeding, and searching via Google (see Table 3). Overall, only one-third of HCPs cited knowing the official website gezondzwangerworden.be; of them, 41% had already referred patients to this website. Furthermore, half of the HCPs (strongly) agreed relying on the product information in the absence of unambiguous information about a medication (53%). Finally, 87% (strongly) agreed that conflicting information on medication safety during pregnancy and breastfeeding impedes their professional duties.

The main ‘Anatomical Therapeutic Chemical’ (ATC) categories HCPs searched for in the last six months regarding utilization during pregnancy/breastfeeding were systemic anti-infectives (75%), respiratory system (70%), alimentary tract and metabolism (63%), dermatologicals (58%), and anti-parasitic products, insecticides, and repellents (49%) (see Supplementary Material Table S4).

HCPs answered mainly searching for information on the risk of a medication for congenital malformations (82%), a safer or better studied medication in pregnancy (76%) or breastfeeding (72%), the amount of medication in breastmilk (67%) and dose adjustments during pregnancy/breastfeeding (66%) (see Table 4 and Supplementary Materials Table S5).

### 3.4. Public’s Information and Counseling Preferences Regarding a Teratology Information Service

Overall, 98% of the public respondents stated being willing to use information on medication use in pregnancy/breastfeeding provided by a future Belgian TIS. Most respondents replied being willing to search for information on the use of temporary medicines (93%), medicines to treat pregnancy-related ailments (86%) or to alleviate ailments after childbirth or during lactation (80%), pregnancy vitamins (78%), and vaccines (76%). Interest in information on supplements (53%) and herbal remedies (51%) was also often noted, especially among French-speaking respondents regarding herbal remedies (77%) (see Supplementary Material Table S6). Next, 94% also cited being willing to use information on non-pharmacological measures (lifestyle/nutrition advice) to avoid/alleviate pregnancy or breastfeeding related ailments. Finally, 95% were positive about using TIS information focusing on the prevention of infections (e.g., cytomegalovirus, toxoplasmosis, varicella, listeria).

With regard to potential distribution channels, respondents generally were in favor of consulting the TIS information on medication use in pregnancy/breastfeeding, non-pharmacological advice, and infection prevention strategies mainly via a website (96–98%) and mobile app (63–66%), and, to a lesser extent, social media (24–30%). While a mobile app was the preferred choice by a quarter of the respondents (25–27%), a website was the most preferred distribution channel (64–69%) (see Supplementary Material Table S7).

Moreover, 78% indicated being willing to contact the TIS in case of personal questions on medication use during pregnancy/breastfeeding. Respondents mainly preferred e-mail (32%), telephone (31%), and live chat (25%). Although one-fifth cited being willing to have a real-life consultation with a TIS expert, only 2% considered this the preferred way of contact (see Supplementary Material Table S8). Women with pregnancy (80% vs. 67%; *p* = 0.05) or breastfeeding experience (80% vs. 70%; *p* = 0.002) and having used medicines during breastfeeding (82% vs. 74%; *p* = 0.02) were more likely to be willing to contact the TIS in case of questions.

The most preferred moments to contact the TIS were weekdays between 18 and 20 h (37%), between 9 and 12 h (19%) and between 13 and 17 h (16%). Half of the respondents wanted to be able to contact the TIS during the weekend (Saturday: 61%; Sunday: 44%) (see Supplementary Material Table S9).

The maximum time respondents found acceptable to wait for a reaction from the TIS was 15 min in case of telephone contact (94%). In addition, 66% replied being OK with waiting at least three hours before receiving a reply via e-mail (and 87% with waiting one hour) (see Supplementary Material Table S10).

Overall, 95% stated having (full) confidence in a TIS regarding information on medication use during pregnancy and breastfeeding. Half of the respondents believed that they would only sometimes discuss the personal information provided by the TIS with any HCP (52%). With regard to personal contribution to research, 90% answered being willing to register individual data on medication use, pregnancy outcomes and infant development.

### 3.5. HCPs’ Information and Counseling Preferences Regarding a Teratology Information Service

Overall, 99% of the HCPs reported being willing to use the evidence-based information on medication and related products during pregnancy/breastfeeding provided by a future Belgian TIS. In fact, 97% of the HCPs (strongly) agreed that their professional activities would benefit from a Belgian database with up-to-date information on medication use during pregnancy/breastfeeding. HCPs expect the TIS to provide information on medications (98%), vaccines (90%), pregnancy vitamins (78%), herbal remedies (76%), supplements (75%) and drugs (75%). Other topics included the risks associated with infections (and how to prevent infections) (69%) and with radiopharmaceuticals/contrast media (62%) (see Supplementary Material Table S11). To consult the information, HCPs indicated preferring the use of a website (80%) or mobile app (14%) (see Supplementary Material Table S12).

Moreover, 92% of the HCPs indicated being willing to contact the TIS in case of specific questions on medication use during pregnancy or breastfeeding. Gender (*p* = 0.44), age (*p* = 0.09) and frequency of contact with the target population (*p*-values ranging between 0.55–0.67) were not associated with willingness to contact the TIS for specific questions. However, willingness slightly differed according to profession: hospital pharmacists: 100%; pediatricians/neonatologists: 96%; lactation consultants: 95%; community pharmacists: 93%; midwives: 93%; GPs: 90%; gynecologists: 78%; and medical specialists: 75%.

HCPs mainly want to contact the TIS for information on the risk of congenital malformations (74%), dose adjustments during pregnancy or breastfeeding (72%), a safer or better-studied medication during pregnancy (71%) or breastfeeding (70%), and the amount of medication in breastmilk (61%) (see Table 4). Overall, 94% of HCPs (strongly) agreed that they would be able to better fulfill their professional activities if the TIS could be contacted in case of specific questions.

To contact the TIS, HCPs mainly preferred the telephone (55%) and e-mail (33%) (see Supplementary Material Table S13). The most preferred times to contact the TIS for (non-urgent) questions were weekdays between 13 and 17 h (40%) and 9 and 12 h (38%). Only few HCPs would like to contact the center during the weekend (Saturday: 15%, Sunday: 5%) (see Supplementary Material Table S14).

The maximum time HCPs found acceptable to wait for a reaction from the TIS was 15 min in case of telephone contact (95%). In addition, 47% replied being OK with waiting at least three hours before receiving a reply via e-mail (and 73% with waiting one hour) (see Supplementary Material Table S15).

Finally, 81% of the HCPs answered being willing to register patient data on medication exposure, pregnancy complications, neonatal outcomes, and infant development, and hence, to contribute to enlarging the amount of safety data on medication use in pregnancy and breastfeeding.

## 4. Discussion

### 4.1. Main Findings

This study aimed to provide insight into the information needs and counseling preferences of the public and HCPs in Belgium regarding medication use in pregnancy/breastfeeding. In fact, this needs assessment involving potential TIS users also provides evidence on their perceptions towards the role of a TIS in the provision of information and counseling, and on operational requirements that should be considered when setting up a TIS.

First, women and HCPs reported high information needs and subsequent seeking behavior regarding medication use during pregnancy and breastfeeding, in line with the findings of previous studies on this topic [9,10,11]. However, most women and HCPs responded as frequently relying on the patient information leaflet (PIL)/product information when searching for information on this topic. HCPs also stated relying on the product information when unambiguous information about a medication is lacking, which is often the case during pregnancy and breastfeeding [1,26]. Nonetheless, previous studies have shown that discrepancies in recommendations for medication use in these patients are omniprevalent between the PIL/product information and evidence-based sources [2,3,4,5,6]. Marketing authorization holders recently also acknowledged their ongoing struggle with providing accurate and timely information on medication safety in pregnancy in the product information [27]. In addition, Google and online fora were found to be other commonly used information sources (or serve as starting point for information searches). This finding deserves attention as many posts on social media and fora may provide inaccurate information [28,29]. In contrast, the evidence-based website on this topic (www.gezondzwangerworden.be (accessed on 19 June 2022)) was only used by a minority of women and was unknown to many HCPs. No improvement in the knowledge or utilization of the website by the public was observed compared to the results of a survey in 2016–2017 [11]. This underscores the need of public campaigns and communication to HCPs to promote the website and, in general, to direct both groups to reliable sources. Strikingly, one-fourth of women declared that their searching activities were the result of conflicting or insufficient information on perinatal medication use received from HCPs, as shown earlier [1,2,3,4,5,6]. Almost all HCPs also agreed that conflicting information on perinatal medication safety complicates their professional duties. These findings underline the importance of the availability of, and easy access of patients and HCPs to, reliable and consistent evidence-based information on medication use in pregnancy/breastfeeding.

Second, most women and HCPs in our cohort replied being willing to use the information provided by a future Belgian TIS on, but not limited to, medication use in pregnancy and breastfeeding. This information should preferably be accessible through a website, and, to a lesser extent, via a mobile app. While women reported trusting this information, HCPs stated that having access to up-to-date information on this topic would positively affect their professional activities. Specific groups of medicines for which HCPs want information were identified, including systemic anti-infectives and respiratory and alimentary tract medicines. These medicines are also regularly used in this population [30,31,32,33,34,35]. Moreover, women and HCPs expressed their interest in having access to information on herbal remedies. This is an interesting and important observation, and somewhat in line with previous research in Belgium showing that 42% of pregnant women prefer to use natural remedies during pregnancy [11]. Nevertheless, there is only limited documentation on the safety of herbal remedies during pregnancy [36], and these remedies should therefore only be used with professional guidance and/or after having read evidence-based information.

Third, a large majority of women and HCPs indicated willingness to personally contact the TIS in case of questions on medication use during pregnancy and breastfeeding. In fact, most HCPs agreed that they would be able to better fulfill their professional activities if the TIS could be contacted in the case of specific questions. The contact with the TIS would preferably occur by phone (especially for HCPs) [20] and e-mail, and during office hours (for HCPs) and in the evening/on the weekend (for women). Small differences were found across disciplines in terms of willingness to contact the TIS. However, given the limited number of participants of some disciplines, no firm conclusions on these differences could be drawn yet, requiring further investigation. In addition, it should be noted that a quarter of the women prefer to chat with the TIS. An online chat service is currently in place in TIS, being part of the American Organization of Teratology Information Specialists (OTIS) and could be a useful tool to extend the outreach of the TIS activities [37]. Moreover, the maximum time most women and HCPs found acceptable to wait for a reaction from the TIS depended on how the center was approached, with, surprisingly, a quarter of the HCPs being willing to receive an e-mail reply within an hour. Finally, half of the women stated that they would only sometimes discuss the information received during personal contact with the TIS with any HCP. This finding is potentially worrisome and a point of attention but is generally in line with previous research showing that less than one-third of pregnant women discussed online-retrieved information with their HCPs [11]. Therefore, it is imperative that TIS staff always recommend patients to discuss the received information with their physicians and/or midwives. This message should also be included on the TIS website along with the information.

To fulfill perinatal medicines’ information needs of patients and HCPs, sufficient safety data are needed, emphasizing the need for reliable data registration and research initiatives. Data registration should preferably occur in close collaboration with patients and HCPs, organized within the healthcare context and performed by using a user-friendly system [27]. Pregnancy exposure and outcome data can be routinely collected by a TIS [17] and used as part of observational cohort studies in collaboration with other centers [38,39,40,41]. Recently, and in line with similar initiatives abroad [42,43], a prospective registry collecting real-world data on medication utilization during pregnancy and mother–infant outcome data has been set-up in Belgium (www.belpreg.be (accessed on 19 June 2022)) and would ideally be run in close collaboration with the future TIS. In that perspective, it is nice to see that more than 80% of women and HCPs reported being willing to register (personal) data on medication exposure, pregnancy and neonatal outcomes and infant development for research purposes.

Finally, most HCPs reported already having searched for information on the amount of medication in breastmilk after maternal exposure, and that they would contact the TIS in the future for this kind of information. This observation underlines the importance of having sufficient safety data available on the transfer of medicines to breastmilk. However, the available data on this subject are currently scarce [44], and for some medicines only consist of (single) case reports or case series with a limited number of included cases [45]. Hence, more attention for clinical lactation studies and non-clinical research methods to enhance our knowledge on this subject is also warranted [26,46,47].

### 4.2. Strengths and Limitations

This study has several strengths. First, a large sample of in total >2000 Dutch/French-speaking women and HCPs of various perinatal disciplines, and often faced with questions on medication use during pregnancy and breastfeeding, was obtained. Enrollment was not restricted to pregnant women and breastfeeding mothers who recently delivered, allowing us to obtain a larger sample and to enhance the validity and robustness of the findings with regard to the perinatal population. The observed high frequency of medication use in pregnancy and in the last 7 days was similar to prevalence rates previously observed in perinatal drug utilization studies in Belgium [32,48]. Second, the high Internet penetration rate in Belgium as well as the anonymous nature of both surveys may have enhanced the correctness of the collected data and data quality. Respondents could answer the questions without the risk of disclosing their identity. Both surveys were also quite similar, allowing us to acquire answers to the same research questions for both groups. Third, this was the first Belgian study exploring information needs and counseling preferences of the public and HCPs, and their perceptions towards the role of a future TIS. Such needs assessment involving all stakeholders and different user perspectives is vital to set up a service that satisfies their needs/desiderata as much as possible.

Some limitations should also be considered. First, an online survey disseminated to the public via social media may entail a risk of selection bias, potentially recruiting a higher proportion of knowledgeable and ‘experienced’ respondents related to the topic of interest. Public survey respondents were higher educated, more often employed and engaged in a relationship [23,24,25]. The lesser involvement of low(er) educated individuals may be in line with the group of people actually contacting a TIS. This should deserve our attention in future studies as well as when implementing the TIS in order to improve equity of access. A German study has indeed shown that high-level educated women tend to be overrepresented among TIS enquirers [49]. French-speaking and male citizens, as well as HCPs of some professions with extensive contact with pregnant and breastfeeding women and recent mothers, i.e., gynecologists, pediatricians/neonatologists, and lactation consultants, participated to a lesser extent, limiting the external validity of the conclusions for these groups. Future research initiatives should focus more on the recruitment of the latter types of professionals, along with HCPs belonging to disciplines with a less intrinsic connection to pregnant and breastfeeding women, to confirm or reject the preliminary observation of small differences across disciplines in willingness to contact the TIS. Second, up to one-quarter of the participants did not finish the survey. It cannot be excluded that the opinions of these respondents somewhat differed, underlining the importance of obtaining insight into the opinion of non-responders in the future and how to engage them with the information and services provided by the TIS. It can also not be excluded that HCPs completed the public survey (as well). Third, almost up to 100% of the respondents declared being willing to use information on medication use provided by a future TIS, impeding the assessment of characteristics associated with (un)willingness. Finally, this quantitative study could not provide answers to some relevant questions. Therefore, qualitative research should be performed to determine factors influencing the utilization of TIS information or its counseling services by users, as well as to explore which experiences would prevent persons from relying again on the TIS information and/or counseling services. In any case, information needs and users’ satisfaction with TIS information and counseling services, as well as characteristics of individuals contacting the TIS, should be closely monitored as soon as a TIS is operational in Belgium.

## 5. Conclusions

Information needs and subsequent seeking behavior regarding medication use during pregnancy and breastfeeding were ubiquitous in this cohort of highly educated and professionally active women and HCPs in Belgium. Despite their limitations, both groups often rely on the patient information leaflet or product information and online fora to find consistent and high-quality information on this topic. Conflicting information on medication safety during pregnancy occurs regularly and complicates HCPs’ professional duties. Both women and HCPs assigned an important role to a Teratology Information Service (TIS) in Belgium to provide evidence-based information on this topic, preferably to be consulted via a website or app. Women and HCPs are also willing to personally contact the TIS in case of questions, preferably by phone or via e-mail, although a relevant proportion of women prefer a chat function. A TIS in Belgium would be warmly welcomed by potential users and should ideally be established soon to address the current information needs and opportunities regarding perinatal medication use and to contribute to research in this field. By aligning the operational aspects of the TIS with stakeholders’ needs and preferences, optimal accessibility, ease of use, and satisfaction with the provided information and services will be achieved.

## Figures and Tables

**Table 3:** Frequency of use by HCPs of some information sources regarding medication use during pregnancy/breastfeeding (N = 606).

	Never	Yearly	Monthly	Weekly	Daily
	% (n)	% (n)	% (n)	% (n)	% (n)
Checking the product information	11.4 (69)	23.1 (140)	39.8 (241)	20.5 (124)	5.3 (32)
Discussing with a colleague from the same profession	16.3 (99)	28.4 (172)	33.3 (202)	17.0 (103)	5.0 (30)
Checking guidelines on treatment of conditions during lactation	24.3 (147)	36.6 (222)	30.4 (184)	8.4 (51)	0.3 (2)
Checking guidelines on treatment of conditions in pregnancy	26.6 (161)	34.8 (211)	30.9 (187)	6.8 (41)	1.0 (6)
Searching via Google	40.8 (247)	22.6 (137)	23.3 (141)	10.7 (65)	2.6 (16)
Discussing with a colleague from another profession	35.8 (217)	37.3 (226)	22.1 (134)	4.1 (25)	0.7 (4)
Visiting online fora	74.3 (450)	10.1 (61)	10.2 (62)	5.0 (30)	0.5 (3)
Visiting websites of perinatal organization(s)	67.3 (408)	19.0 (115)	10.9 (66)	2.8 (17)	0.0 (0)
Contacting a pharmaceutical company	71.9 (436)	24.9 (151)	2.5 (15)	0.7 (4)	0.0 (0)
Requesting information from patient associations	95.4 (578)	2.3 (14)	1.7 (10)	0.5 (3)	0.2 (1)

Results are shown as % (n).

**Table 4:** Type of information HCPs search for and for which they would contact the future Teratology Information Service.

Type of Information	Type of Information I Am Usually Looking for (N = 565)	Type of Information I Would Contact the TIS about (N = 500)
	% (n)	% (n)
Risk of a medication for congenital malformations	82.3 (465)	73.6 (368)
A safer or better studied medication during pregnancy	75.9 (429)	71.2 (356)
A safer or better studied medication during breastfeeding	72.0 (407)	70.4 (352)
The amount of medication in breast milk	66.9 (378)	60.8 (304)
Dose adjustments during pregnancy or breastfeeding	66.2 (374)	72.0 (360)
Pediatric usage or dosage of a medication	55.6 (314)	41.4 (207)
How to treat pregnancy-related ailments	52.4 (296)	35.6 (178)
Effect of a medication on pregnancy outcomes (e.g., low birth weight)	51.0 (288)	56.2 (281)
Information about vaccines during pregnancy/breastfeeding	48.1 (272)	52.0 (260)
Effect of a medication on (future) child development (e.g., IQ, autism, …)	42.5 (240)	54.0 (270)

Results are shown as % (n). Only the ten most frequently cited examples of information HCPs are usually looking for are presented here. A complete overview can be found in the Supplementary Material Table S5. HCPs = healthcare professionals.

## Data Availability

Not applicable.

## Supplementary Materials

The following supporting information can be downloaded at: https://www.mdpi.com/article/10.3390/ijerph19148605/s1; Table S1: Overview of the frequency of the contact of participating HCPs with the target population; Table S2: Information sources used by the public with regard to medication use in general, during pregnancy and breastfeeding; Table S3: Potential reasons reported by the public for searching information on medication use in general & during pregnancy/breastfeeding; Table S4: Types of medication already searched for by HCPs regarding use in pregnancy/breastfeeding, according to ATC level 1; Table S5: Type of information HCPs search for and for which they would contact the future Teratology Information Service in Belgium; Table S6: Preferences of the public regarding the utilization of information provided by the future Belgian Teratology Information Service; Table S7: Preferences of the public regarding different channels to distribute information by the Teratology Information Service in Belgium; Table S8: Preferences of the public with regard to contacting the future Teratology Information Service in Belgium in case of personal questions on medication use during pregnancy or breastfeeding; Table S9: Preferences of the public regarding the timing to contact the future Teratology Information Service in Belgium in case of personal questions on medication use during pregnancy or breastfeeding; Table S10: Preferences of the public regarding the acceptable ‘waiting’ time when contacting the Belgian Teratology Information Service; Table S11: Preferences of HCPs regarding the availability of information provided by the future Belgian Teratology Information Service; Table S12: Preferences of HCPs regarding different distribution channels to consult information provided by the future Teratology Information Service in Belgium; Table S13: Preferences of HCPs with regard to contacting the future Teratology Information Service in Belgium in case of specific questions on medication use during pregnancy or breastfeeding; Table S14: Preferences of HCPs regarding the timing to contact the future Teratology Information Service in Belgium in case of specific (non-urgent) questions on medication use during pregnancy or breastfeeding; Table S15: Preferences of HCPs towards the acceptable ‘waiting’ time when contacting the future Teratology Information Service in Belgium.
