# Supplementary material for: Information Needs and Counseling Preferences among Potential Users of the Future Teratology Information Service in Belgium: A Cross-Sectional Study Involving the Public and Healthcare Professionals
Source: Int J Environ Res Public Health. 2022 Jul 14;19(14):8605. doi: 10.3390/ijerph19148605 (PMC9319400; doi:10.3390/ijerph19148605)
Supplement: Supplementary file 1 [file ijerph-19-08605-s001.zip › ijerph-1802955-supplementary.pdf]

International Journal of
Environmental Research and Public Health

MDPI

Supplementary Materials

Table S1. Overview of the frequency of the contact of participating healthcare professionals with the target population (*N* = 532).

Frequency of Contact with	Never	Yearly	Monthly	Weekly	Daily
	% (*n*)	% (*n*)	% (*n*)	% (*n*)	% (*n*)
Women/couples willing to get pregnant	13.0 (69)	19.7 (105)	29.9 (159)	24.8 (132)	12.6 (67)
Pregnant women	4.9 (26)	10.0 (53)	22.9 (122)	35.7 (190)	26.5 (141)
Breastfeeding women	3.8 (20)	10.3 (55)	25.0 (133)	29.3 (156)	31.6 (168)
(Nursing) infants	13.0 (69)	5.5 (29)	14.1 (75)	31.8 (169)	35.7 (190)

Results are shown as % (*n*).

Table S2. Information sources used by the public with regard to medication use in general, during pregnancy and breastfeeding.

Source of Information	Medication Use in General			Medication Use during Pregnancy			Medication Use during Breastfeeding		
	Total	NL	FR	Total	NL	FR	Total	NL	FR
	(*N* = 1264)	(*N* = 983)	(*N* = 281)	(*N* = 1208)	(*N* = 939)	(*N* = 269)	(*N* = 873)	(*N* = 644)	(*N* = 229)
Patient information leaflet	**94.8 (1198)**	94.7 (931)	95.0 (267)	**90.2 (1090)**	89.7 (842)	92.2 (248)	**86.5 (755)**	85.4 (550)	89.5 (205)
Google or other search engine(s)	**83.5 (1055)**	83.3 (819)	84.0 (236)	**76.6 (925)**	77.4 (727)	73.6 (198)	**69.6 (608)**	69.6 (448)	69.9 (160)
Website/social media of an (online) pharmacy	**23.8 (301)**	23.3 (229)	25.6 (72)	**15.0 (181)**	15.5 (146)	13.0 (35)	**11.9 (104)**	12.7 (82)	9.6 (22)
Scientific resources	**22.5 (285)**	19.5 (192)	33.1 (93)	**22.4 (271)**	21.5 (202)	25.7 (69)	**22.6 (197)**	21.4 (138)	25.8 (59)
Online fora	**19.5 (246)**	18.7 (184)	22.1 (62)	**25.2 (304)**	25.1 (236)	25.3 (68)	**23.3 (203)**	23.0 (148)	24.0 (55)
Website/social media of a hospital	**15.7 (199)**	18.7 (184)	5.3 (15)	**13.3 (161)**	15.3 (144)	6.3 (17)	**8.2 (72)**	9.6 (62)	4.4 (10)
Website/social media of a health insurance agency	**12.3 (156)**	14.4 (142)	5.0 (14)	**8.7 (105)**	10.4 (98)	2.6 (7)	**6.2 (54)**	7.8 (50)	1.7 (4)
Website/social media of the government	**9.9 (125)**	10.0 (98)	9.6 (27)	**6.7 (81)**	7.0 (66)	5.6 (15)	**4.8 (42)**	4.3 (28)	6.1 (14)
Website/social media of a healthcare professional	**4.9 (62)**	4.1 (40)	7.8 (22)	**5.5 (67)**	4.9 (46)	7.8 (21)	**6.0 (52)**	5.4 (35)	7.4 (17)
A foreign website	**4.1 (52)**	2.7 (27)	8.9 (25)	**3.6 (43)**	2.7 (25)	6.7 (18)	**6.4 (56)**	5.4 (35)	9.2 (21)
Via contact with the National Poison Centre	**3.2 (40)**	2.8 (28)	4.3 (12)	**0.7 (8)**	0.3 (3)	1.9 (5)	**0.9 (8)**	0.3 (2)	2.6 (6)
Via patient associations	**2.8 (35)**	3.0 (29)	2.1 (6)	**3.4 (41)**	3.8 (36)	1.9 (5)	**3.0 (26)**	2.8 (18)	3.5 (8)
Via contact with a pharmaceutical company	**1.3 (16)**	1.0 (10)	2.1 (6)	**0.9 (11)**	0.7 (7)	1.5 (4)	**0.8 (7)**	0.8 (5)	0.9 (2)
Website/social media of perinatal organization(s)	**N/A**	N/A	N/A	**21.0 (254)**	25.5 (239)	5.6 (15)	**20.8 (182)**	25.6 (165)	7.4 (17)

Results are shown as % (*n*). The results are ordered in descending order for the information sources most frequently used by the public related to searching for information on medication use in general. N/A = not applicable. NL = Dutch-speaking public respondents. FR = French-speaking public respondents.

Table S3. Potential reasons reported by the public for searching information on medication use in general & during pregnancy/breastfeeding.

Potential Reason	Medication Use in General			Medication Use during Pregnancy/Breastfeeding		
	**Total (*N* = 1264)**	**NL (*N* = 983)**	**FR (*N* = 281)**	**Total (*N* = 1267)**	**NL (*N* = 977)**	**FR (*N* = 290)**
To have more information on potential side effects.	**63.3 (800)**	63.3 (622)	63.3 (178)	**37.8 (479)**	37.4 (365)	39.3 (114)
To check the received information.	**57.7 (729)**	54.6 (537)	68.3 (192)	**49.8 (631)**	46.8 (457)	60.0 (174)
To facilitate making decisions about personal health.	**46.5 (588)**	43.5 (428)	56.9 (160)	**34.1 (432)**	31.0 (303)	44.5 (129)
To have more information on how the medication works.	**43.3 (547)**	45.9 (451)	34.2 (96)	**21.6 (274)**	20.7 (202)	24.8 (72)
To have more information on how long the medication can be used.	**35.5 (449)**	38.4 (377)	25.6 (72)	**21.9 (278)**	24.0 (234)	15.2 (44)
Having received insufficient information from a healthcare professional.	**34.4 (436)**	30.4 (299)	48.8 (137)	**25.3 (320)**	20.8 (203)	40.3 (117)
Having received unclear information from a healthcare professional.	**27.8 (351)**	24.4 (240)	39.5 (111)	**21.2 (269)**	17.2 (168)	34.8 (101)
Having received conflicting information from healthcare professionals.	**24.8 (314)**	22.0 (216)	34.9 (98)	**26.5 (336)**	22.7 (222)	39.3 (114)
To be prepared for a conversation with a healthcare professional.	**14.2 (180)**	12.8 (126)	19.2 (54)	**13.2 (167)**	11.6 (113)	18.6 (54)
Not having had enough time during the consultation with a healthcare professional.	**13.3 (168)**	10.8 (106)	22.1 (62)	**6.4 (81)**	4.4 (43)	13.1 (38)
Not having dared to ask the healthcare professional (additional) questions.	**10.2 (129)**	8.0 (79)	17.8 (50)	**5.5 (70)**	4.6 (45)	8.6 (25)
To have more information on the safety of a medication for the fetus/nursing infant.	**N/A**	N/A	N/A	**67.2 (851)**	69.8 (682)	58.3 (169)
To have more information on the safety of a medication for herself (i.e., as a pregnant or breastfeeding woman).	**N/A**	N/A	N/A	**53.8 (682)**	53.9 (527)	53.4 (155)

Results are shown as % (*n*). The results are ordered in descending order for the reasons most frequently reported related to searching for information on medication use in general. N/A = not applicable. NL = Dutch speaking public respondents. FR = French speaking public respondents.

Table S4. Types of medication already searched for by HCPs regarding use in pregnancy/breastfeeding, according to ATC level 1.

ATC Level 1	Pregnancy			Breastfeeding			Haven't Searched for Any Information regarding Pregnancy/Breastfeeding		
	TOT (N = 579)	NL (N = 380)	FR (N = 199)	TOT (N = 579)	NL (N = 380)	FR (N = 199)	TOT (N = 579)	NL (N = 380)	FR (N = 199)
Anti-infectives for systemic use	**63.7** **(369)**	62.1 (236)	66.8 (133)	**61.1** **(354)**	61.1 (232)	61.3 (122)	**24.7** **(143)**	24.7 (94)	24.6 (49)
Respiratory system	**58.7** **(340)**	60.0 (228)	56.3 (112)	**54.4** **(315)**	53.7 (204)	55.8 (111)	**30.4** **(176)**	29.7 (113)	31.7 (63)
Alimentary tract and metabolism (including insulin)	**54.2** **(314)**	56.1 (213)	50.8 (101)	**43.2** **(250)**	42.6 (162)	44.2 (88)	**37.1** **(215)**	35.8 (136)	39.7 (79)
Dermatologicals	**49.1** **(284)**	49.7 (189)	47.7 (95)	**43.5** **(252)**	42.9 (163)	44.7 (89)	**41.6** **(241)**	41.1 (156)	42.7 (85)
Anti-parasitic products, insecticides, and repellents	**44.0** **(255)**	42.6 (162)	46.7 (93)	**39.6** **(229)**	37.1 (141)	44.2 (88)	**50.8** **(294)**	52.6 (200)	47.2 (94)
Nervous system	**37.7** **(218)**	32.9 (125)	46.7 (93)	**33.3** **(193)**	29.7 (113)	40.2 (80)	**54.7** **(317)**	59.2 (225)	46.2 (92)
Cardiovascular system	**35.4** **(205)**	36.1 (137)	34.2 (68)	**24.5** **(142)**	24.5 (93)	24.6 (49)	**54.9** **(318)**	53.7 (204)	57.3 (114)
Genito-urinary system and sex hormones	**35.8** **(207)**	35.0 (133)	37.2 (74)	**31.8** **(184)**	32.1 (122)	31.2 (62)	**55.1** **(319)**	54.5 (207)	56.3 (112)
Sensory organs	**28.7** **(166)**	30.0 (114)	26.1 (52)	**25.4** **(147)**	25.3 (96)	25.6 (51)	**67.2** **(389)**	65.8 (250)	69.8 (139)
Systemic hormonal preparations, excluding sex hormones and insulins	**22.6** **(131)**	25.8 (98)	16.6 (33)	**21.9** **(127)**	25.0 (95)	16.1 (32)	**68.9** **(399)**	64.2 (244)	77.9 (155)
Musculo-skeletal system	**25.4** **(147)**	18.7 (71)	38.2 (76)	**22.1** **(128)**	16.3 (62)	33.2 (66)	**69.8** **(404)**	77.1 (293)	55.8 (111)
Blood and blood forming organs	**23.5** **(136)**	30.8 (117)	9.5 (19)	**14.9** **(86)**	19.5 (74)	6.0 (12)	**72.5** **(420)**	63.9 (243)	88.9 (177)
Antineoplastic and immunomodulating agents	**10.4** **(60)**	11.8 (45)	7.5 (15)	**10.0** **(58)**	10.8 (41)	8.5 (17)	**86.9** **(503)**	85.5 (325)	89.4 (178)

Results are shown as % (n). HCP = healthcare professional. NL = Dutch-speaking HCPs. FR = French-speaking HCPs. The results show the percentage of HCPs who have searched, in the last six months, for any information on medication use during pregnancy / breastfeeding. Medications were categorized according to the Anatomical Therapeutic Chemical (ATC) level 1 classification. The sum of the total percentages for each ATC level (i.e., sum of grey columns) exceed 100% as HCPs could have searched both for information on medication use in pregnancy and breastfeeding.

Table S5. Type of information HCPs search for and for which they would contact the future Teratology Information Service in Belgium.

Type of Information	Type of Information I'm Usually Looking for			Type of Information I Would Contact the TIS about		
	Total (*N* = 565)	NL (*N* = 371)	FR (*N* = 194)	Total (*N* = 500)	NL (*N* = 328)	FR (*N* = 172)
Risk of a medication for congenital malformations	**82.3 (465)**	80.1 (297)	86.6 (168)	**73.6 (368)**	71.0 (233)	78.5 (135)
A safer or better studied medication during pregnancy	**75.9 (429)**	78.4 (291)	71.1 (138)	**71.2 (356)**	71.0 (233)	71.5 (123)
A safer or better studied medication during breastfeeding	**72.0 (407)**	74.9 (278)	66.5 (129)	**70.4 (352)**	71.0 (233)	69.2 (119)
The amount of medication in breast milk	**66.9 (378)**	69.5 (258)	61.9 (120)	**60.8 (304)**	60.7 (199)	61.0 (105)
Dose adjustments during pregnancy or breastfeeding	**66.2 (374)**	67.4 (250)	63.9 (124)	**72.0 (360)**	70.4 (231)	75.0 (129)
Pediatric usage or dosage of a medication	**55.6 (314)**	52.8 (196)	60.8 (118)	**41.4 (207)**	36.6 (120)	50.6 (87)
How to treat pregnancy-related ailments	**52.4 (296)**	51.2 (190)	54.6 (106)	**35.6 (178)**	31.4 (103)	43.6 (75)
Effect of a medication on pregnancy outcomes (e.g., low birth weight)	**51.0 (288)**	52.0 (193)	49.0 (95)	**56.2 (281)**	53.7 (176)	61.0 (105)
Information about vaccines during pregnancy/breastfeeding	**48.1 (272)**	47.2 (175)	50.0 (97)	**52.0 (260)**	51.2 (168)	53.5 (92)
Effect of a medication on (future) child development (e.g., IQ, autism, …)	**42.5 (240)**	36.7 (136)	53.6 (104)	**54.0 (270)**	49.1 (161)	63.4 (109)
How to treat breastfeeding-related ailments	**41.9 (237)**	41.8 (155)	42.3 (82)	**40.0 (200)**	36.3 (119)	47.1 (81)
How to treat pregnancy-related conditions	**38.2 (216)**	42.3 (157)	30.4 (59)	**36.2 (181)**	34.8 (114)	39.0 (67)
How to treat a chronic condition during pregnancy	**31.3 (177)**	27.8 (103)	38.1 (74)	**41.2 (206)**	38.1 (125)	47.1 (81)
How to treat a chronic condition during breastfeeding	**31.0 (175)**	29.1 (108)	34.5 (67)	**42.0 (210)**	39.0 (128)	47.7 (82)
Preconception advice for a medication	**29.7 (168)**	32.3 (120)	24.7 (48)	**39.2 (196)**	37.8 (124)	41.9 (72)
Frequency and/or type of neonatal withdrawal symptoms	**20.2 (114)**	20.2 (75)	20.1 (39)	**38.2 (191)**	35.4 (116)	43.6 (75)
Recommendation for more comprehensive ultrasound examinations due to exposure to a medication	**6.7 (38)**	7.0 (26)	6.2 (12)	**24.8 (124)**	25.9 (85)	22.7 (39)
Information about radiopharmaceuticals and contrast media	**N/A**	N/A	N/A	**33.6 (168)**	33.5 (110)	33.7 (58)

Results are shown as % (*n*). N/A = not available. TIS = Teratology Information Service. HCPs = healthcare professionals. NL = Dutch-speaking HCPs. FR = French-speaking HCPs.

Table S6. Preferences of the public regarding the utilization of information provided by the future Belgian Teratology Information Service.

Type of Information	Total (*N* = 1163)	NL (*N* = 920)	FR (*N* = 243)
Information on medicines that I need to use temporarily (e.g., as a result of an infection).	**93.1** **(1083)**	92.7 (853)	94.7 (230)
Information on medicines used to treat pregnancy-related ailments (e.g., nausea and vomiting, heartburn, constipation, hemorrhoids).	**85.6** **(996)**	86.5 (796)	82.3 (200)
Information on medicines used to alleviate ailments after childbirth or during lactation (e.g., sore nipples, hemorrhoids, episiotomy).	**79.5** **(925)**	78.4 (721)	84.0 (204)
Information on pregnancy vitamins.	**77.9** **(906)**	80.1 (737)	69.5 (169)
Information on vaccines (e.g., influenza vaccine, pertussis ('whooping cough') vaccine, …).	**75.6** **(879)**	76.4 (703)	72.4 (176)
Information on medicines to treat pregnancy-related conditions (e.g., gestational diabetes, preeclampsia, …).	**64.0** **(744)**	64.0 (589)	63.8 (155)
Information on medicines I have to use chronically.	**57.9** **(673)**	56.0 (515)	65.0 (158)
Information on (nutritional) supplements.	**52.8** **(614)**	52.5 (483)	53.9 (131)
Information on herbal/natural remedies.	**51.4** **(598)**	44.7 (411)	77.0 (187)

Results are shown as % (*n*). The results show the percentage of public respondents who responded being interested in utilizing the specific type of information, if provided by the future Teratology Information Service in Belgium. NL = Dutch-speaking public respondents. FR = French-speaking public respondents.

Table S7. Preferences of the public regarding different channels to distribute information by the Teratology Information Service in Belgium.

	Topic 1: Information on Medication Use during Pregnancy and Breastfeeding						Topic 2: Information on Non-Pharmacological Measures (i.e., Lifestyle and Nutrition Advice)						Topic 3: Information on the Prevention of Infections (e.g., Cytomegalovirus, Toxoplasmosis, Varicella, Listeriosis…)					
Distribution Channel	Willing to Consult the Information via This Channel			Preferred Way to Consult the Information			Willing to Consult the Information via This Channel			Preferred Way to Consult the Information			Willing to Consult the Information via This Channel			Preferred Way to Consult the Information		
	Total (*N* = 1158)	NL (*N* = 918)	FR (*N* = 240)	Total (*N* = 1152)	NL (*N* = 915)	FR (*N* = 237)	Total (*N* = 1094)	NL (*N* = 867)	FR (*N* = 227)	Total (*N* = 1090)	NL (*N* = 867)	FR (*N* = 223)	Total (*N* = 1098)	NL (*N* = 881)	FR (*N* = 217)	Total (*N* = 1095)	NL (*N* = 880)	FR (*N* = 215)
A website	**98.4**	98.5	98.3	**69.1**	71.0	61.6	**95.9**	95.7	96.5	**63.9**	65.4	57.8	**95.9**	96.1	94.9	**66.2**	67.7	60.0
	**(1140)**	(904)	(236)	**(796)**	(650)	(146)	**(1049)**	(830)	(219)	**(696)**	(567)	(129)	**(1053)**	(847)	(206)	**(725)**	(596)	(129)
A mobile application	**65.5**	65.9	64.2	**26.3**	24.7	32.5	**63.7**	63.8	63.4	**26.5**	25.4	30.9	**62.9**	62.9	63.1	**25.1**	24.4	27.9
	**(759)**	(605)	(154)	**(303)**	(226)	(77)	**(697)**	(553)	(144)	**(289)**	(220)	(69)	**(691)**	(554)	(137)	**(275)**	(215)	(60)
Social Media	**23.9**	24.0	23.8	**2.4**	2.5	2.1	**30.4**	30.4	30.4	**5.8**	5.7	6.3	**27.9**	27.8	28.1	**3.9**	3.5	5.6
	**(277)**	(220)	(57)	**(28)**	(23)	(5)	**(333)**	(264)	(69)	**(63)**	(49)	(14)	**(306)**	(245)	(61)	**(43)**	(31)	(12)
A newsletter	**9.8**	9.5	10.8	**1.0**	0.9	1.3	**12.2**	12.9	9.7	**1.7**	1.8	1.3	**11.9**	12.7	8.8	**1.8**	1.9	1.4
	**(113)**	(87)	(26)	**(11)**	(8)	(3)	**(134)**	(112)	(22)	**(19)**	(16)	(3)	**(131)**	(112)	(19)	**(20)**	(17)	(3)
Flyers	**11.1**	10.8	12.5	**0.6**	0.3	1.7	**10.4**	10.3	11.0	**0.5**	0.3	1.3	**14.1**	13.7	15.7	**1.5**	1.5	1.4
	**(129)**	(99)	(30)	**(7)**	(3)	(4)	**(114)**	(89)	(25)	**(6)**	(3)	(3)	**(155)**	(121)	(34)	**(16)**	(13)	(3)
A magazine	**7.5**	7.0	9.6	**0.3**	0.3	0.4	**8.8**	7.7	12.8	**1.1**	1.0	1.3	**8.8**	7.7	13.4	**1.0**	0.6	2.8
	**(87)**	(64)	(23)	**(4)**	(3)	(1)	**(96)**	(67)	(29)	**(12)**	(9)	(3)	**(97)**	(68)	(29)	**(11)**	(5)	(6)
A blog	**4.7**	4.8	4.6	**0.3**	0.2	0.4	**9.9**	9.6	11.0	**0.5**	0.3	0.9	**8.2**	8.1	8.8	**0.5**	0.3	0.9
	**(55)**	(44)	(11)	**(3)**	(2)	(1)	**(108)**	(83)	(25)	**(5)**	(3)	(2)	**(90)**	(71)	(19)	**(5)**	(3)	(2)

Results are shown as % (*n*). NL = Dutch-speaking public respondents. FR = French-speaking public respondents. The results show the percentage of the public respondents willing to consult and pre-

ferring to consult the information on medication use in pregnancy/breastfeeding, non-pharmacological measures, and infection prevention strategies, as provided by the future Teratology Information Service in Belgium, according to potential distribution channels.

Table S8. Preferences of the public with regard to contacting the future Teratology Information Service in Belgium in case of personal questions on medication use during pregnancy or breastfeeding.

Potential Ways to Contact the TIS	Willing to Contact the TIS in This Way			Preferred Way to Contact the TIS		
	Total (*N* = 889)	NL (*N* = 684)	FR (*N* = 205)	Total (*N* = 887)	NL (*N* = 684)	FR (*N* = 203)
Through e-mail	**82.0 (729)**	82.7 (566)	79.5 (163)	**31.9 (283)**	31.6 (216)	33.0 (67)
Over the telephone	**67.3 (598)**	63.0 (431)	81.5 (167)	**30.8 (273)**	27.8 (190)	40.9 (83)
Through live chat on the center's website	**62.1 (552)**	63.3 (433)	58.0 (119)	**25.5 (226)**	28.2 (193)	16.3 (33)
Through WhatsApp	**36.1 (321)**	37.3 (255)	32.2 (66)	**9.5 (84)**	10.0 (68)	7.9 (16)
During a real-life consultation	**18.9 (168)**	18.0 (123)	22.0 (45)	**1.7 (15)**	1.9 (13)	1.0 (2)
I would ask the center to call me at a certain time	**9.6 (85)**	9.2 (63)	10.7 (22)	**0.7 (6)**	0.6 (4)	1.0 (2)

Results are shown as % (*n*). TIS = Teratology Information Service. NL = Dutch-speaking public respondents. FR = French-speaking public respondents.

Table S9. Preferences of the public regarding the timing to contact the future Teratology Information Service in Belgium in case of personal questions on medication use during pregnancy or breastfeeding.

Timing to Contact the TIS	Willing to Contact the TIS			Preferred Time to Contact the TIS		
	Total (*N* = 889)	NL (*N* = 684)	FR (*N* = 205)	Total (*N* = 889)	NL (*N* = 684)	FR (*N* = 205)
During weekdays between 18 h and 20 h	**71.9 (639)**	73.7 (504)	65.9 (135)	**37.0 (329)**	39.6 (271)	28.3 (58)
During weekdays between 9 h and 12 h	**66.7 (593)**	65.6 (449)	70.2 (144)	**18.8 (167)**	17.5 (120)	22.9 (47)
During weekdays between 13 h and 17 h	**65.2 (580)**	64.9 (444)	66.3 (136)	**16.0 (142)**	14.3 (98)	21.5 (44)
During weekdays between 16 h and 18 h	**62.5 (556)**	64.5 (441)	56.1 (115)	**11.4 (101)**	12.4 (85)	7.8 (16)
On Saturday	**61.3 (545)**	59.6 (408)	66.8 (137)	**5.8 (52)**	5.6 (38)	6.8 (14)
During weekdays between 12 h and 13 h	**52.2 (464)**	52.9 (362)	49.8 (102)	**5.1 (45)**	5.0 (34)	5.4 (11)
On Sunday	**44.3 (394)**	41.7 (285)	53.2 (109)	**3.0 (27)**	2.5 (17)	4.9 (10)
During weekdays between 8 h and 9 h	**45.0 (400)**	44.7 (306)	45.9 (94)	**2.9 (26)**	3.1 (21)	2.4 (5)

Results are shown as % (*n*). TIS = Teratology Information Service. NL = Dutch-speaking public respondents. FR = French-speaking public respondents.

Table S10. Preferences of the public regarding the acceptable 'waiting' time when contacting the Belgian Teratology Information Service.

Acceptable Waiting Time for a Reaction from the TIS	Over the Telephone			Via E-Mail			Via Live Chat			Via WhatsApp			In Case the TIS Will Call Me back at a Later Time		
	**Total (*N* = 878)**	**NL (*N* = 678)**	**FR (*N* = 200)**	**Total (*N* = 878)**	**NL (*N* = 678)**	**FR (*N* = 200)**	**Total (*N* = 878)**	**NL (*N* = 678)**	**FR (*N* = 200)**	**Total (*N* = 878)**	**NL (*N* = 678)**	**FR (*N* = 200)**	**Total (*N* = 878)**	**NL (*N* = 678)**	**FR (*N* = 200)**
<5 min	**53.6 (471)**	56.2 (381)	45.0 (90)	**1.1 (10)**	1.0 (7)	1.5 (3)	**41.7 (366)**	42.6 (289)	38.5 (77)	**17.7 (155)**	16.2 (110)	22.5 (45)	**11.0 (97)**	5.2 (35)	36.0 (62)
5–15 min	**40.1 (352)**	37.8 (256)	48.0 (96)	**1.8 (16)**	1.8 (12)	2.0 (4)	**35.4 (311)**	36.1 (245)	33.0 (66)	**29.8 (262)**	28.0 (190)	36.0 (72)	**9.2 (81)**	4.4 (30)	25.5 (51)
15–30 min	**3.6 (32)**	3.4 (23)	4.5 (9)	**3.0 (26)**	2.1 (14)	6.0 (12)	**9.3 (82)**	9.6 (65)	8.5 (17)	**12.2 (107)**	13.1 (89)	9.0 (18)	**4.4 (39)**	3.4 (23)	8.0 (16)
30–60 min	**0.8 (7)**	0.7 (5)	1.0 (2)	**7.3 (64)**	6.8 (46)	9.0 (18)	**5.4 (47)**	4.6 (31)	8.0 (16)	**14.0 (123)**	14.6 (99)	12.0 (24)	**7.1 (62)**	7.4 (50)	6.0 (12)
1–3 h	**1.3 (11)**	1.5 (10)	0.5 (1)	**20.5 (180)**	18.6 (126)	27.0 (54)	**4.7 (41)**	4.4 (30)	5.5 (11)	**13.4 (118)**	13.3 (90)	14.0 (28)	**22.9 (201)**	26.0 (176)	12.5 (25)
3–24 h	**0.5 (4)**	0.3 (2)	1.0 (2)	**42.4 (372)**	43.5 (295)	38.5 (77)	**3.0 (26)**	2.1 (14)	6.0 (12)	**10.1 (89)**	11.7 (79)	5.0 (10)	**30.5 (268)**	35.5 (241)	13.5 (27)
>24 h	**0.1 (1)**	0.1 (1)	0.0 (0)	**23.9 (210)**	26.3 (178)	16.0 (32)	**0.6 (5)**	0.6 (4)	0.5 (1)	**2.7 (24)**	3.1 (21)	1.5 (3)	**14.8 (130)**	18.1 (123)	3.5 (7)

Results are shown as % (*n*). TIS = Teratology Information Service. NL = Dutch-speaking public respondents. FR = French-speaking public respondents.

Table S11. Preferences of HCPs regarding the availability of information provided by the future Belgian Teratology Information Service.

Type of Information	Total (*N* = 551)	NL (*N* = 366)	FR (*N* = 185)
Information about medications	**98.2 (541)**	98.4 (360)	97.8 (181)
Information about vaccines	**89.8 (495)**	91.3 (334)	87.0 (161)
Information about pregnancy vitamins	**78.4 (432)**	80.6 (295)	74.1 (137)
Information about herbal/natural remedies	**76.2 (420)**	72.1 (264)	84.3 (156)
Information about (nutritional) supplements	**75.3 (415)**	77.9 (285)	70.3 (130)
Information about the risk and effects of drugs	**74.6 (411)**	72.7 (266)	78.4 (145)
Information about the risks and how to prevent infections	**69.0 (380)**	69.9 (256)	67.0 (124)
Information about radiopharmaceuticals and contrast media	**61.9 (341)**	61.5 (225)	62.7 (116)

Results are shown as % (*n*). HCPs = healthcare professionals. TIS = Teratology Information Service. NL = Dutch-speaking HCPs. FR = French-speaking HCPs. The results show the percentage of HCPs replying that the future TIS in Belgium should make this type of information available.

Table S12. Preferences of HCPs regarding different distribution channels to consult information provided by the future Teratology Information Service in Belgium.

Distribution Channel	Willing to Consult the Information via This Channel			Preferred Way to Consult the Information		
	Total (*N* = 549)	NL (*N* = 364)	FR (*N* = 185)	Total (*N* = 539)	NL (*N* = 358)	FR (*N* = 181)
A website	**96.9 (532)**	98.4 (358)	94.1 (174)	**80.0 (431)**	83.2 (298)	73.5 (133)
A mobile application	**55.0 (302)**	60.2 (219)	44.9 (83)	**13.9 (75)**	13.7 (49)	14.4 (26)
A newsletter	**26.4 (145)**	23.6 (86)	31.9 (59)	**3.9 (21)**	1.7 (6)	8.3 (15)
A magazine	**12.9 (71)**	13.7 (50)	11.4 (21)	**1.9 (10)**	1.1 (4)	3.3 (6)
Social Media	**6.4 (35)**	6.6 (24)	5.9 (11)	**0.4 (2)**	0.3 (1)	0.6 (1)
A blog	**0.5 (3)**	0.5 (2)	0.5 (1)	**0.0 (0)**	0.0 (0)	0.0 (0)

Results are shown as % (*n*). HCPs = healthcare professionals. TIS = Teratology Information Service. NL = Dutch-speaking HCPs. FR = French-speaking HCPs. The results show the percentage of HCPs willing to consult and preferring to consult the information on the use of medication and related products during pregnancy and breastfeeding, as provided by the future Teratology Information Service in Belgium, and according to potential distribution channels.

Table S13. Preferences of HCPs with regard to contacting the future Teratology Information Service in Belgium in case of specific questions on medication use during pregnancy or breastfeeding.

Potential Ways to Contact the TIS	Willing to Contact the TIS in This Way			Preferred Way to Contact the TIS		
	Total (*N* = 514)	NL (*N* = 334)	FR (*N* = 180)	Total (*N* = 512)	NL (*N* = 333)	FR (*N* = 179)
Over the telephone	**86.0 (442)**	84.4 (282)	88.9 (160)	**55.1 (282)**	52.6 (175)	59.8 (107)
Through e-mail	**82.7 (425)**	82.3 (275)	83.3 (150)	**33.4 (171)**	35.4 (118)	29.6 (53)
Through live chat on the center's website	**22.6 (116)**	26.0 (87)	16.1 (29)	**6.8 (35)**	7.8 (26)	5.0 (9)
Through WhatsApp	**17.3 (89)**	18.9 (63)	14.4 (26)	**3.7 (19)**	3.3 (11)	4.5 (8)
I would ask the center to call me at a certain time	**3.5 (18)**	4.2 (14)	2.2 (4)	**1.0 (5)**	0.9 (3)	1.1 (2)

Results are shown as % (*n*). HCPs = healthcare professionals. TIS = Teratology Information Service. NL = Dutch-speaking HCPs. FR = French-speaking HCPs.

Table S14. Preferences of HCPs regarding the timing to contact the future Teratology Information Service in Belgium in case of specific (non-urgent) questions on medication use during pregnancy or breastfeeding.

Timing to Contact the TIS	Willing to Contact the TIS			Preferred Time to Contact the TIS		
	Total (*N* = 514)	NL (*N* = 334)	FR (*N* = 180)	Total (*N* = 514)	NL (*N* = 334)	FR (*N* = 180)
During weekdays between 13 h and 17 h	**81.9 (421)**	83.5 (279)	78.9 (142)	**39.9 (205)**	38.9 (130)	41.7 (75)
During weekdays between 9 h and 12 h	**83.3 (428)**	82.9 (277)	83.9 (151)	**38.1 (196)**	37.4 (125)	39.4 (71)
During weekdays between 12 h and 13 h	**36.6 (188)**	39.8 (133)	30.6 (55)	**7.8 (40)**	9.9 (33)	3.9 (7)
During weekdays between 16 h and 18 h	**41.1 (211)**	43.4 (145)	36.7 (66)	**5.4 (28)**	6.0 (20)	4.4 (8)
During weekdays between 18 h and 20 h	**21.0 (108)**	22.2 (74)	18.9 (34)	**4.7 (24)**	3.9 (13)	6.1 (11)
During weekdays between 8 h and 9 h	**27.4 (141)**	28.1 (94)	26.1 (47)	**2.5 (13)**	2.4 (8)	2.8 (5)
On Saturday	**15.0 (77)**	13.2 (44)	18.3 (33)	**1.4 (7)**	1.2 (4)	1.7 (3)
On Sunday	**5.4 (28)**	5.7 (19)	5.0 (9)	**0.2 (1)**	0.3 (1)	0.0 (0)

Results are shown as % (*n*). HCPs = healthcare professionals. TIS = Teratology Information Service. NL = Dutch-speaking HCPs. FR = French-speaking HCPs.

Table S15. Preferences of HCPs towards the acceptable 'waiting' time when contacting the future Teratology Information Service in Belgium.

Acceptable Waiting Time for a Reaction from the TIS	Over the Telephone			Via E-Mail			Via Live Chat			Via WhatsApp			In Case the TIS Will Call Me back at a Later Time		
	Total (*N* = 510)	NL (*N* = 331)	FR (*N* = 179)	Total (*N* = 510)	NL (*N* = 331)	FR (*N* = 179)	Total (*N* = 510)	NL (*N* = 331)	FR (*N* = 179)	Total (*N* = 510)	NL (*N* = 331)	FR (*N* = 179)	Total (*N* = 510)	NL (*N* = 331)	FR (*N* = 179)
<5 min	**75.1 (383)**	77.6 (257)	70.4 (126)	**3.1 (16)**	1.5 (5)	6.1 (11)	**47.3 (241)**	49.2 (163)	43.6 (78)	**24.5 (125)**	20.2 (67)	32.4 (58)	**15.1 (77)**	4.8 (16)	34.1 (61)
5–15 min	**20.2 (103)**	16.9 (56)	26.3 (47)	**4.7 (24)**	3.3 (11)	7.3 (13)	**24.9 (127)**	26.9 (89)	21.2 (38)	**21.6 (110)**	23.6 (78)	17.9 (32)	**7.1 (36)**	2.4 (8)	15.6 (28)
15–30 min	**1.8 (9)**	1.8 (6)	1.7 (3)	**6.7 (34)**	5.4 (18)	8.9 (16)	**6.5 (33)**	4.5 (15)	10.1 (18)	**12.4 (63)**	12.1 (40)	12.8 (23)	**6.3 (32)**	5.4 (18)	7.8 (14)
30–60 min	**0.8 (4)**	0.9 (3)	0.6 (1)	**12.9 (66)**	12.1 (40)	14.5 (26)	**6.3 (32)**	6.3 (21)	6.1 (11)	**13.1 (67)**	14.2 (47)	11.2 (20)	**11.6 (59)**	12.4 (41)	10.1 (18)
1–3 h	**0.8 (4)**	0.9 (3)	0.6 (1)	**25.7 (131)**	24.8 (82)	27.4 (49)	**5.7 (29)**	4.8 (16)	7.3 (13)	**14.5 (74)**	16.3 (54)	11.2 (20)	**25.9 (132)**	30.8 (102)	16.8 (30)
3–24 h	**1.2 (6)**	1.5 (5)	0.6 (1)	**37.3 (190)**	42.3 (140)	27.9 (50)	**4.9 (25)**	4.8 (16)	5.0 (9)	**9.2 (47)**	10.0 (33)	7.8 (14)	**24.9 (127)**	32.9 (109)	10.1 (18)
>24 h	**0.2 (1)**	0.3 (1)	0.0 (0)	**9.6 (49)**	10.6 (35)	7.8 (14)	**4.5 (23)**	3.3 (11)	6.7 (12)	**4.7 (24)**	3.6 (12)	6.7 (12)	**9.2 (47)**	11.2 (37)	5.6 (10)

Results are shown as % (*n*). HCPs = healthcare professionals. TIS = Teratology Information Service. NL = Dutch-speaking HCPs. FR = French-speaking HCPs.
